# TAT and HA2 Facilitate Cellular Uptake of Gold Nanoparticles but Do Not Lead to Cytosolic Localisation

**DOI:** 10.1371/journal.pone.0121683

**Published:** 2015-04-02

**Authors:** Yann Cesbron, Umbreen Shaheen, Paul Free, Raphaël Lévy

**Affiliations:** 1 Department of Chemistry, University of Liverpool, Liverpool, United Kingdom; 2 Institute of Integrative Biology, University of Liverpool, Liverpool, United Kingdom; 3 CNRS, UMR 6290, Institute of Genetics and Development of Rennes, Rennes, France; 4 Université de Rennes 1, Université Européenne de Bretagne, Structure fédérative de recherche Biosit, Faculté de Médecine, Rennes, France; 5 Institute of Materials Research and Engineering, A*STAR, 3 Research Link, Singapore, Singapore; Universidade Nova de Lisboa, PORTUGAL

## Abstract

The methods currently available to deliver functional labels and drugs to the cell cytosol are inefficient and this constitutes a major obstacle to cell biology (delivery of sensors and imaging probes) and therapy (drug access to the cell internal machinery). As cell membranes are impermeable to most molecular cargos, viral peptides have been used to bolster their internalisation through endocytosis and help their release to the cytosol by bursting the endosomal vesicles. However, conflicting results have been reported on the extent of the cytosolic delivery achieved. To evaluate their potential, we used gold nanoparticles as model cargos and systematically assessed how the functionalisation of their surface by either or both of the viral peptides TAT and HA2 influenced their intracellular delivery. We evaluated the number of gold nanoparticles present in cells after internalisation using photothermal microscopy and their subcellular localisation by electron microscopy. While their uptake increased when the TAT and/or HA2 viral peptides were present on their surface, we did not observe a significant cytosolic delivery of the gold nanoparticles.

## Introduction

To gain access to the interior of cells, molecules and particles need to cross the cell membrane. As the lipid bilayer is impermeable to polar molecules, intracellular transport normally relies on specific transmembrane protein channels/transporters or endocytotic processes. The ability to deliver probes or drugs with preserved functions to the cell cytosol would therefore have a large impact in cell biology, drug delivery and ultimately therapy. Despite reports suggesting that some nanomaterials can simply cross the cell membrane, very little convincing experimental evidence of direct cytosolic entry exists in the literature [1,2]. Eukaryotic cells exposure to nanomaterials such as gold nanoparticles tends to result in their uptake through various endocytotic processes. This has been known for over 5O years: one of the earliest application of gold nanoparticles in biology was precisely as a tracer to understand the potential mechanisms of entry of viruses inside cells and whether membranous structures observed in the cytosol derived from the cell membrane [3]. The entry mechanism is directly related to physicochemical properties of the bioconjugates, *e*.*g*. overall charge, size, peptide- or protein-binding capacity[4,5]. Gold nanoparticles are mainly localised in endolysosomes after their uptake by cells [6–10], and remain sequestered in these intracellular compartments thereafter. Furthermore, engineered multifunctional surface coating molecules, such as nanoparticle-anchored peptides and proteins, are susceptible to degradation from proteases inside the vesicular compartments [10]. Two consequences arise from the entrapment: particle surface functions may be compromised and particle free diffusion within the cytosol or nucleus is blocked. As a result, interactions of the particles with other cell components are prevented. Two main routes have been explored to bypass entrapment: either a direct one through the membrane (direct translocation, microinjection, electroporation or bacterial pore forming toxins) or an indirect one via endocytosis followed by endosome lysis (osmotic shock or drug induced). These methods have flaws, among which their low throughput, invasiveness and cytotoxicity [7,11–13]. Some have been highly disputed [14–17]. Therefore improving the efficiency of delivery of nanomaterials targeted to the cell cytosol by avoiding or disrupting the endosomal entrapment remains a major challenge. Conjugation with cell-penetrating peptides (CPPs) is an internalisation strategy that has been explored and successfully applied to a variety of cargoes in the past two decades [18], including several types of nanoparticles [19–22]. Likewise, the membrane lysis capabilities of fusion peptides can be exploited to achieve a release from the endolysosomes. The viral peptides TAT and HA2 can be used in combination for intracellular delivery of nanomaterials. The former (CPP) will bolster endocytosis-driven intracellular transport and the latter (fusion peptide) will promote a release to the cytosol. Because of their straightforward synthesis, surface biofunctionalisation and interesting physicochemical properties, gold nanoparticles have been proposed as ideal vehicles or probes for intracellular delivery [23–26]. However, evidences for targeted cytosolic or nuclear localisation of gold particles using TAT and HA2 peptides remain limited and the conclusions of different reports appear to be conflicting [27–33]. The TAT peptide, from the transcription activation factor of the human immunodeficiency virus, is arginine-rich and was one of the first reported CPPs [34]. Its cellular uptake and further nuclear localisation were first observed by two independent groups in 1988 [35,36]. TAT was initially thought to translocate through the cell membrane, but macropinocytosis was later identified as the main entry mechanism [37–40]. HA2 is a subunit of the influenza A virus hemagglutinin glycoprotein HA. It includes a hydrophobic fusion peptide sequence that promotes lipid membrane destabilisation at low pH via a N-terminus insertion into the membrane [41,42].

Wadia *et al*. reported on the influence of both TAT and HA2 on cargo delivery by comparing the influence of dTAT, dHA2 and dTAT-HA2 peptides on the delivery efficiency of the TAT peptide fused to the Cre recombinase (TAT-Cre) in tex.loxP.EG cells [38]. The peptide dTAT-HA2 improved the delivery of TAT-Cre to the nucleus, while dTAT had a marginally positive effect and dHA2 a neutral one. The advantage of their methodology lies in conclusively assessing the nuclear delivery of the non-fluorescent Cre recombinase through measurements of fluorescence signal resulting from eGFP expression induced by Cre. However, gene expression (after sequence deletion) does not necessitate a large number of delivered functions to operate and the authors noted that the majority of the TAT-Cre peptide remained trapped in macropinosomes. Nevertheless, building on this report and on an article later retracted [43], Kumar *et al*. described the use of HA2-TAT peptides to deliver multifunctional gold nanoparticles as imaging agents in live cells [44]. A combination of TAT-HA2, PEGs and anti-actin antibodies were used to functionalise the surface of gold cores. Although delivery within cells of gold cores was established (TEM), the interpretation is rendered difficult by the anti-actin antibody attachment strategy (acid-labile hydrazone bond are susceptible to cleavage during endocytosis [45]). In the context of protein cargo delivery, Sugita *et al*. suggested that HA2-TAT peptides can increase the cytosolic delivery of protein-fused TAT, but do not enhance the protein internalisation [39]. Todorava *et al*. reported an increase in internalisation of peptide-capped gold nanoparticles presenting the TAT sequence [46]. Others have used polyarginines (4–12 repeats) rather than TAT to provide CPP properties. Michiue *et al*. observed an increased transcriptional activity of HA2 fused p53-9R compared to p53-11R, causing an enhanced antitumor activity of HA2-p53-9R as compared to p53-11R alone [40]. A careful assessment of the effect of each and every part of a delivery system is important. Indeed, while studying the complex internalisation mechanisms of the Alexa488-labelled polyarginine R12, Hirose *et al*. revealed that the dye also played a role in the internalisation of the conjugate R12-Alexa488 [47]. Likewise, Liou *et al*. established that both the CPP and fusion part of the R9-HA2-mCherry construct had an effect on its uptake kinetics and efficiency. They also showed that part of the endocytosis-internalised constructs remained in endolysosomes [48]. A report by Lee *et al*. employed the HA2 analogue E5 and TAT peptides co-incubated with fluorescein-labelled dextrans, which indicated that E5 remained associated with endosomal membranes even in the case of lysis evidenced by a cytosolic distribution of dextran [49].

To clarify the potential of TAT and HA2 peptides (alone or in combination), and building on our prior work on peptide-capped gold nanoparticles [50,51], we have prepared a variety of conjugates and used photothermal microscopy and electron microscopy to detect the quantity of internalised HA2 and/or TAT functionalised gold nanoparticles and their localisation within the cells.

## Materials and Methods

### Materials

Tissue culture medium was from Gibco Life Technologies (Carlsbad, CA, USA); foetal calf serum (FCS) from Harlan Sera-Lab (UK). Gold nanoparticles were synthesised as mentioned further. Peptides used were from Anaspec (San Jose, CA, USA) and Peptides Proteins Research Ltd (Fareham, UK). The peptides CCALNN-HA2, HA2-NNLACC, CCALNN-dHA2, CALNN and CCALNN-PEG (see Table 1 for sequences) were purchased from Peptides Proteins Research Ltd (UK). Thiol-PEG (HS-(CH_2_)_11_-(ethylene glycol)_4_-glycinol, Mol. Wt. 380.58 g.mol^-1^) was from ProChimia Surfaces (Poland). Osmium tetroxide (4% aqueous solution) was from TAAB Laboratories Equipment Ltd (UK) (diluted in water to 1% v/v). Uranyl acetate powder and lead nitrate were from Agar Scientific (UK) and sodium citrate from Sigma-Aldrich (USA). The 16% paraformaldehyde solution (diluted to 4% v/v) was from Agar Scientific (UK) and the 25% glutaraldehyde solution (diluted to 2.5% v/v) from TAAB Laboratories Equipment Ltd (UK). 30 kDa Nanosep centrifugation filters for nanoparticle purification and sterilisation were bought from VWR (USA) and PALL (USA). 35mm glass bottom coverslip dishes were from Iwaki (Japan) and MatTek Corporation (USA) and 35mm cell culture glass bottom (10mm) coverslip dishes from Corning (USA). Tween 20 and Sephadex G-25 beads for size exclusion chromatography were from Sigma Aldrich (USA).

**Table 1:** Nomenclature of the peptides utilised, with the short names and corresponding amino acid sequences.

Sequence No.	Peptide name	Peptide sequence
1	CCALNN–dHA2	CCALNNGdimGewGneifGaiaGflG-NH_2_
2	HA2–NNLACC	GLFEAIEGFIENGWEGMIDGWYGGGGNNLACC
3	CCALNN–HA2	CCALNNGGGGLFEAIEGFIENGWEGMIDGWYG
4	CALNN–TAT	CALNNAGRKKRRQRRR
5	CCALNN–PEG	CCALNN-(ethylene glycol)_6_–glycinol
6	CALNN	CALNN

### Peptide stock solutions

**Fig 1:**
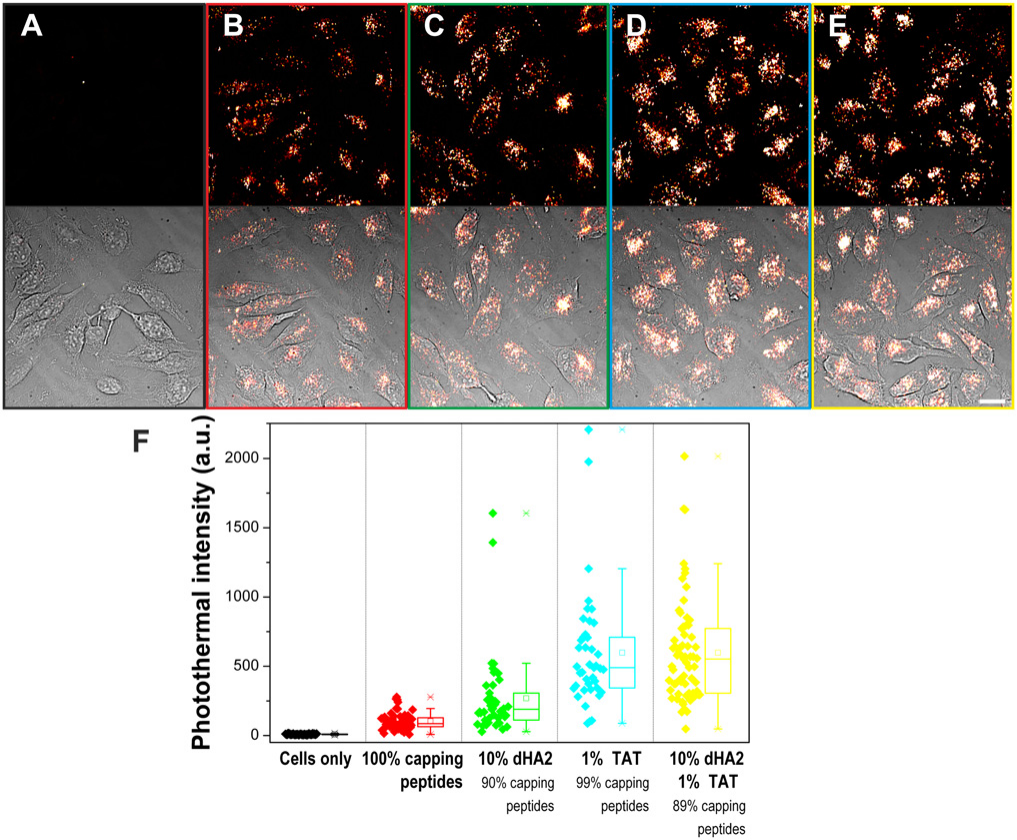
Intracellular delivery of gold nanoparticles assisted by monolayer functionalisation with HA2/TAT peptides. HeLa cells were incubated with/without 5nm nanoparticles (500nM) functionalised with either capping peptides (CALNN: CCALNN-PEG at a 4:1 molar ratio) or with a combination of dHA2 and/or TAT functional peptides with capping peptides for 3h, fixed and imaged by photothermal microscopy. (A-E) Images of the nanoparticles uptake by HeLa cells, scale bar represents 20μm. Top: photothermal image; bottom: overlay of bright field and photothermal images. (A) Cells without nanoparticles. (B) Cells incubated with CALNN: CCALNN-PEG 4:1 coated nanoparticles. (C) Cells incubated with 10% CCALNN-dHA2 coated nanoparticles. (D) Cells incubated with 1% CALNN-TAT coated nanoparticles. (E) Cells incubated with 10% CCALNN-dHA2 + 1% CALNN-TAT coated nanoparticles. (F) Quantification of the internalisation of gold nanoparticles for the conditions shown in (A-E), measured via the mean photothermal intensities of individual cells (over 40 cells for each condition, each data point represents one cell). All conditions are significantly different from one another except the pair (1% TAT, 10% dHA2-1% TAT). Dataset available on figshare (10.6084/m9.figshare.1088379).

Full peptides sequences are shown in Table 1. Stock solutions (2mM) of CALNN, CCALNN-PEG, HA2-NNLACC and CCALNN-HA2 peptides were prepared by dissolving the peptides in concentrated phosphate buffer saline (10x PBS: 1.6M NaCl, 30mM KCl, 80mM Na_2_HPO_4_, 10mM KH_2_PO_4_). The stock solution of the peptide CCALNN-dHA2 was prepared by dissolving it in dimethyl-sulfoxide (DMSO) at 2mM. The stock solutions of the peptide CALNN-TAT were prepared by dissolving it in either 10x PBS at 2nM (Fig. 1) or in dimethyl-sulfoxide (DMSO) at 0.7mM (S4 Fig). The resulting solutions were kept as aliquots at −80°C.

### Gold nanoparticle synthesis

Nanoparticle sizes refer to their diameter. For 5 and 10nm gold nanoparticles, the methods were adapted and modified from Slot *et al*. [52] and from Kimling *et al*. [53]. For 5 nm gold colloid, 395mL of deionised water and 5mL of 1% w/v HAuCl_4_ was heated to 60°C. Then a 100mL solution containing 20mL 1% w/v tri-sodium citrate, 6mL 1% w/v tannic acid, 6mL 0.2M KCO_3_ and 68mL of deionised water was added and left to stir at 60°C to complete the reaction. For 10nm, the concentration of tannic acid was reduced (0.002% w/v final concentration) and no KCO_3_ was added. For 15nm gold nanoparticles, the citrate reduction method was adapted and modified from Frens [54]. 50mL of 0.01% HAuCl_4_ was gently heated and stirred to boiling. Then 1.25 mL of 1% tri-sodium citrate was added to the solution. Continuous heating and stirring was maintained until a deep red wine colour had appeared. The solution was then stirred for another 10–15 minutes to complete the reaction.

### Formation of peptide self-assembled monolayers

For each nanoparticle self-assembled monolayer (SAM), a peptide solution was prepared by mixing the peptide stock solutions in the appropriate volume ratio. The peptide stock solution was mixed in a 1:1 ratio with 10x PBS (1.6M NaCl, 30mM KCl, 80mM Na_2_HPO_4_, 10mM KH_2_PO_4_). The resulting peptide solution was added to the gold nanoparticles suspension in a 1:9 volume ratio yielding a final peptide concentration of 100μM. The solution was briefly agitated before addition of Tween 20 to a final concentration of 0.05% (v/v). Formation of the monolayer was immediately visible because of the increased colloidal stability and of a small red shift of the nanoparticles plasmon band (S1 Fig). Higher proportions of TAT in the monolayer resulted in nanoparticle aggregation and therefore were not used for further studies (S1 Fig). The solutions were left overnight at room temperature. Surface compositions were varied by adjusting the peptide mixture compositions. SAM percentage composition refers to the peptide mixture percentage composition (mole/mole) used during SAM formation.

### Nanoparticles purification procedure

The 10nm biofunctionalised gold nanoparticle suspensions were centrifuged (13000 rpm, 60 min), and the pellet was resuspended in 1mL of phosphate buffer saline (PBS: 160mM NaCl, 3mM KCl, 8mM Na_2_HPO_4_, 1mM KH_2_PO_4_) with 0.05% Tween 20 before a second centrifugation step (13000 rpm, 60 min). This purification step was repeated three times with PBS, before resuspension in PBS. The repeated centrifugation procedure ensures that the concentration of excess free peptide used during SAM formation is brought down to the picomolar range. For 5nm biofunctionalised gold nanoparticles, the particles were concentrated using 30 kDa Nanosep centrifugation filters or centrifuged for 2h and then passed through a G-25 size exclusion chromatography column to remove the unbound ligand fraction. The particles were sterilised using 0.22μm centrifugation filters.

### Cell culture and nanoparticles incubation

HeLa cells (Human cervical carcinoma, ECACC No. 93021013) were grown in Dulbecco's modified Eagle medium (DMEM) supplemented with 10% FCS (v/v) and 1% non-essential amino acids (v/v), at 37°C, 5% CO_2_. Cells (between passages 8 to 20) were seeded at 4 x10^5^ cells/dish in 35mm glass bottom coverslip cell culture dishes (Iwaki, Japan; Corning, USA). For all experiments, nanoparticles were incubated directly into the complete medium (containing 10% FCS) for the indicated times and at the stated final concentration.

### Transmission electron microscopy

Cells were seeded at 4 x10^5^ cells per dish in a 35mm Petri dish. One day after plating, the cells were incubated for the indicated time with nanoparticles at a 6nM final concentration. After one to two washes with warm PBS (5 min for each wash), the cells were fixed for 1h using 4% paraformaldehyde and 2.5% glutaraldehyde in a 0.1M phosphate buffer solution, osmicated and processed for epoxy resin embedding. After 48 h of resin embedding, 50–70nm sections were cut with a LKB Ultramicrotome and post-stained with an aqueous 5% uranyl acetate and 2% lead citrate solution. Lead citrate was made from lead nitrate and sodium citrate following Reynold’s recipe [55]. The water used to produce lead citrate was highly purified deionised water (18.2 MOhm), boiled for 10 min to remove CO_2_ and cooled for 30 min (covered). The lead citrate solution was stored in a foil-lined plastic container at 4°C, and centrifuged in Eppendorf tubes at 5000 rpm for 5 min just before use. Electron micrographs were acquired with a FEI Tecnai Spirit (FEI Company, USA) transmission electron microscope.

### Electron microscopy images analysis

**Fig 2:**
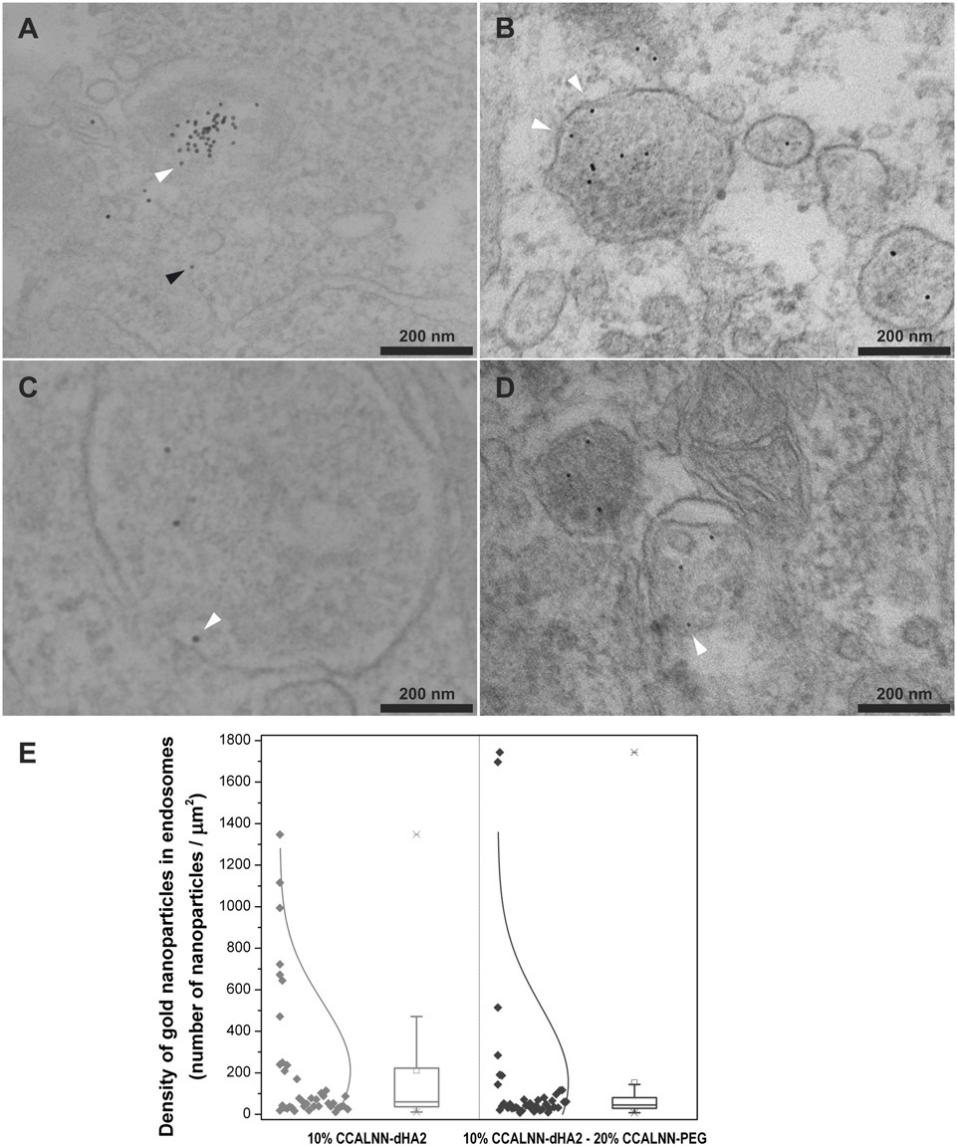
Effect of PEG on the intracellular localisation of HA2 fusion peptide functionalised gold nanoparticles. HeLa cells were incubated with peptide-coated 10nm gold nanoparticles (6nM) for 4h, washed thoroughly with PBS, fixed and imaged by electron microscopy. Nanoparticle peptide monolayer composition: (A-B) 10% CCALNN-dHA2 and 90% CALNN, (C-D) 10% CCALNN-dHA2, 20% CCALNN-PEG and 70% CALNN. Arrowheads point toward gold nanoparticles either interacting with vesicular membranes (white), or displaying a cytosolic localisation (black). (E) Density of nanoparticles in endosomes estimated from images shown in (A-B), n = 30; and (C-D), n = 29. The sets of images analysed as well as additional images are available on figshare (10.6084/m9.figshare.875584, 10.6084/m9.figshare.875630).

**Fig 3:**
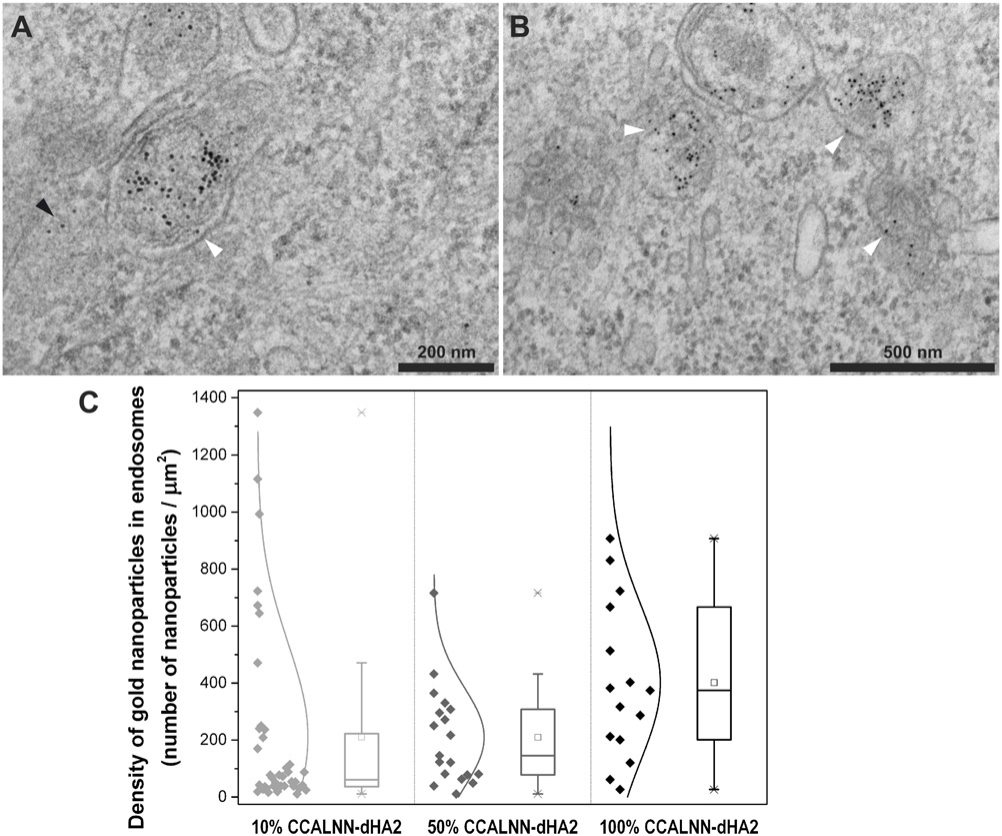
Influence of the proportion of CCALNN-dHA2 peptides in the monolayer of gold nanoparticles. HeLa cells were incubated for 4h with 10nm gold nanoparticles (6nM) coated with a monolayer composed of two different proportions of the peptides CCALNN-dHA2 and CALNN, washed thoroughly with PBS, fixed and imaged by electron microscopy. (A-B) TEM images of the cells incubated with nanoparticle with the following peptide monolayer composition: (A) 50% CCALNN-dHA2 and 50% CALNN, (B) 100% CCALNN-dHA2. Arrowheads point toward gold nanoparticles either interacting with vesicular membranes (white) or displaying a cytosolic localisation (black). (C) Density of nanoparticles in endosomes estimated from images shown in (A) (n = 10, middle) and (B) (n = 10, right); quantification for 10% CCALNN-dHA2 (left) is the same as in Fig. 2E (left). The sets of images analysed are available on figshare (10.6084/m9.figshare.875545, 10.6084/m9.figshare.875477).

In order to estimate the effect of CCALNN-dHA2 density in the SAM on the uptake of gold nanoparticles, the mean number of gold nanoparticles per unit area of endosome was calculated. For each nanoparticle-containing endosome present on a given image, a region of interest (ROI) was drawn around the endosome and the number of gold nanoparticles within the ROI was counted. This number was then divided by the surface area of the ROI to obtain the density of gold nanoparticle in that particular endosome. Measurements were repeated for each nanoparticle-containing endosome and for each image analysed. For 10% CCALNN-dHA2-capped gold nanoparticles, 29 and 30 images were analysed, respectively for nanoparticles with and without CCALNN-PEG (Fig. 2E). 10 images each were analysed for 50% and 100% CCALNN-dHA2-capped gold nanoparticles (Fig. 3C).

### Photothermal microscopy

A frequency-doubled Nd:YLF 523nm laser (Laser Quantum, UK) modulated by an acousto-optic modulator (Isomet Corporation, USA) at a frequency of 692.5kHz was used as heating beam (gold nanoparticle surface plasmon band: ~520nm for 10nm diameter) and a 632.8nm HeNe laser (JDS Uniphase Corporation, USA) was used to probe the time-dependent changes in refractive index induced locally around the nano-objects. The laser beams were focused through a 50x/0.9NA Achroplan oil objective (Zeiss, Germany), and carefully co-aligned to overlap their point-spread functions on the same plane onto the sample. Laser powers were measured before microscope entry and multiplied by a measured transmission coefficient (0.44 at 523nm, 0.57 at 633nm) to evaluate the power at the objective output. Laser powers of 0.44mW and 10.03–10.65mW at the objective output were used, respectively for the heating and probe beams. A 40×/0.8NA Achroplan water-dipping long working distance (2.5mm) objective (Zeiss, Germany) was used to collect the forward scattered field, which was detected by a fast photodiode (New Focus, USA). Images were acquired by raster scanning the sample across the stationary lasers focused 1μm above the glass coverslip where the sample was deposited using a P-563.3CD piezoelectric stage and an E710.3CD controller (Physik Instrumente, Germany). Photothermal signal was integrated over 10ms per pixel (620nm width). A lock-in amplifier was used to extract the signal at the beat note frequency (photothermal signal), which was acquired by a NI PCIe-6259 acquisition card (National Instruments, USA). Hardware equipment and signal acquisition were controlled by a custom-built software developed in Labview. See S2 Fig. for a detailed scheme of the photothermal microscope system used here. The custom-built transmitted light illumination system used an X-Cite Series 120 (EXFO UK, UK) excitation source collimated onto the sample (glass bottom coverslip plastic dish) through an EXFO microscope adaptor, an additional lens (400mm focal length, Thorlabs, USA), and the Achroplan 40×/0.8NA water-dipping objective. The Achroplan 50×/0.9NA oil objective was used to collect the transmitted light. The bright field images (16-bit resolution, 512 × 512 pixels full frame size) were acquired using the HCImage 1.1.1.0 image acquisition software (Hamamatsu, Japan) onto an ImagEM air-cooled EMCCD camera (Hamamatsu, Japan) fastened on the bottom port of an inverted Axiovert 200 microscope (Zeiss, Germany).

### Photothermal image analysis

The analysis was processed using ImageJ (NIH, USA). The bright field and photothermal images, both encoded in 16-bit TIFF file formats, were joined in a TIFF image file composed of two image planes (bright field, photothermal). As the photothermal images were of a 256 × 256 pixels size a two times scaling was applied using ImageJ scaling function with no interpolation. Regions of interest (ROIs) were drawn around each of the cells that were appearing as whole in a given field of view using the bright field image and the freehand image tool of ImageJ. A few regions were drawn on the image using the oval drawing tool of ImageJ in areas where no cells were present. The mean photothermal intensities of these regions were used to produce an average photothermal background intensity. The mean photothermal intensity of each of the cell ROIs was calculated after subtraction of the average photothermal background intensity of the field of view, to yield mean photothermal intensities for each whole cell. This procedure was repeated for each of the images acquired for a given condition and for each condition.

### Statistical analysis and representations

Statistical non-parametric tests were used to assess statistical significance after normality tests showed either non-uniform normality between samples (Figs. 1 & 3) or uniform non-normal distributions (Fig. 2). Kruskal-Wallis tests of variances were used to assess differences between populations, while Kolmogorov-Smirnov pair tests or Mann-Whitney pair tests were used to assess differences between pairs of distributions. A difference between two samples was considered statistically significant when below 5%. Boxplots edges were plotted from the first to the third quartile, with whiskers at distances of 1.5 times the interquartile range away from the edges of the box, dashes representing extrema and crosses the 1^st^ and 99^th^ percentiles. The distributions were computed with ten bins, with a normal distribution fitting curve (Figs. 2 & 3).

## Results and Discussion

### Facilitated uptake of gold nanoparticles by HA2 and TAT peptides

The impact of the cell penetrating peptide TAT and the endosome-disrupting peptide HA2 on the intracellular delivery of gold nanoparticles was quantified by photothermal microscopy (Fig. 1). This absorption-based technique was chosen for its ability to visualise the uptake and intracellular localisation of gold nanoparticles internalised in cells. It allows for the tracking and direct far-field imaging of a range of nanomaterials such as gold [10,56,57], silver [58], and iron oxide nanoparticles [59], single-walled carbon nanotubes [60,61] and quantum dots [62]. Gold nanoparticles (5nm diameter) were functionalised with a self-assembled monolayer composed of the capping peptides CALNN and CCALNN-PEG and a small proportion of either one or both of the functional peptides CALNN-TAT and CCALNN-dHA2 (for peptide sequences, see Table 1). The mixture of CALNN and CCALNN-PEG peptides (4:1 ratio) was selected to constitute the base of the ligand shell, with CALNN providing a dense and compact peptide monolayer [50], while poly(ethylene glycol) (PEG) helps to reduce non-specific interactions [63,64]. Indeed, increasing the proportion of PEG-terminated peptides in the protective monolayer leads to a reduced internalisation of gold nanoparticles (S3 Fig., dataset available on figshare [65]). We hypothesized that limiting nanoparticle non-specific interactions with the plasma membrane should accentuate the difference in uptake between particles bearing functional peptides (TAT and/or HA2) and those that do not, consequently facilitating investigating the role of these functional peptides in the internalisation process. HeLa cells were incubated for 4h with the nanoparticles, fixed and imaged by photothermal microscopy. The intensity of the photothermal signal scales with the quantity of gold nanoparticles internalised into the cells (Fig. 1B-E, dataset available on figshare [66]). The uptake of nanoparticles bearing 1% TAT peptide (Fig. 1D) or 1% TAT and 10% HA2 in their monolayer (Fig. 1E) is substantially higher than that of particles only coated with CALNN and CCALNN-PEG peptides (Fig. 1B). Indeed, nanoparticles comprising 1% TAT peptides within their monolayer display a near six fold increased uptake (Fig. 1F). Further study would be necessary to unambiguously determine the molecular basis for this increased uptake although both the literature [67] and our experiments with both slightly different TAT functionalised gold nanoparticles (S5 Fig) and FITC-TAT peptides (S4 Fig and further discussion in S1 File) suggest that the main cell entry mechanism is macropinocytosis.

Noticeably, the presence of the fusion peptide HA2 in the nanoparticle monolayer also increased the uptake in the absence of TAT by more than two times as compared to nanoparticles with CALNN and CCALNN-PEG peptides only (Fig. 1F). It is therefore reasonable to hypothesise that HA2 peptides display some degree of interaction with the cell membrane during the uptake. The combination of both HA2 and TAT functions had no additive effect in terms of quantity of uptake, when compared to nanoparticles bearing only TAT. The analysis of the variances of the populations, exemplified in Fig. 1A-E, showed a statistically significant difference between them (non-parametric Kruskal-Wallis test). Further series of two-sample tests (non-parametric Kolmogorov-Smirnov tests) showed that only the two samples functionalised with TAT peptides (1% TAT and 10% HA2-1% TAT) are not significantly different from one another (p<2.10^-6^ for the other pairs of sample). Nanoparticles comprising functional peptides (HA2 or/and TAT) or not in their monolayer all display a similar endosomal localisation within the cells, which is visible in the images acquired by photothermal microscopy (Fig. 1B-E). HA2 does not seem to have the anticipated effect of helping the nanoparticle escape the endosomes and disperse in the cytosol. However, the HA2 and/or TAT-terminated peptides clearly facilitated the cellular uptake of gold nanoparticles. The fate of internalised HA2 functionalised-nanoparticles was further studied by transmission electron microscopy (TEM). While TEM cannot be performed on live cells, it has the spatial resolution required to visualise both the electron-dense nanoparticles and the cell organelles, such as endolysosomes, that are not detectable by photothermal microscopy.

### Localisation of internalised HA2-capped nanoparticles: influence of nanoparticle PEGylation

Based on the literature, the hypothesised mode of action of HA2 was that after nanoparticle internalisation via endocytosis, HA2 would interact with the vesicle membrane upon acidification. Poly(ethylene glycol) on gold nanoparticles reduces non-specific interactions, but it could also prevent HA2 insertion into the endosomal membrane, or hinder the membrane destabilisation function. Hence, the influence of PEG-terminated peptides in the monolayer of HA2-functionalised gold nanoparticles on interactions with cell membranes was examined by TEM. Two self-assembled monolayer compositions were compared. Both types of monolayers comprised 10% CCALNN-dHA2 fusion peptides, with or without the addition of 20% CCALNN-PEG peptides, and were completed by CALNN peptides. The gold cores diameter selected were of ~10nm to facilitate unambiguous TEM observation. As previously, HeLa cells were incubated for 4h with the nanoparticles (6nM). The cells were then fixed and processed for TEM observation. In both conditions, gold nanoparticles were mainly confined inside intact or partially ruptured endosomes (Fig. 2). Similarly, nanoparticles were interacting with the inner membrane of intact endosomes (white arrowheads in Fig. 2), regardless of the presence or absence of PEG-terminated peptides within their monolayer. Some particles escaped the endosomal compartments (black arrowhead in Fig. 2A), but most of them remained in or around the disrupted membranes, apparently interacting with them. Only on rare occasions could one observe nanoparticles free in the cytosol (datasets available on figshare [68,69]) irrespective of the presence or absence of PEG within the peptide monolayer. The presence or absence of PEG at the surface of HA2-functionalised gold nanoparticles does not seem to influence their interaction with vesicular membranes. The intracellular localisation for both types of gold nanoparticles remains almost exclusively endosomal, which is in agreement with the photothermal microscopy observations (Fig. 1).

To further infer if subtle differences could be identified between particles bearing or not PEG terminated-peptides, the density of nanoparticle within endosomes (number of particles per unit area of endosome) was quantified from the TEM images. Although Fig. 2A-B suggests that nanoparticles without PEG have a slightly more spread distribution of density in endosomes, a non-parametric statistical analysis of the two populations (Kruskal-Wallis test of variances) and their distributions (Kolmogorov-Smirnov two-sample test) indicates that differences between the two are not statistically significant (p = 0.16 and p = 0.31, respectively). This observation points toward a dominant effect of the HA2 peptides present in the monolayer over the remainder constituents, and a strong interaction with the vesicle membrane.

### Localisation of internalised HA2-capped nanoparticles: effect of HA2 grafting density

As shown by photothermal microscopy, the addition of HA2-terminated peptides in the peptide self-assembled monolayer of gold nanoparticles increases the uptake of these nanoparticles (Fig. 1). The particles are internalised by endocytosis and do not show a cytosolic localisation, remaining essentially in endosomes. It is therefore reasonable to assume that HA2 peptides display some degree of interaction with the cell membrane during the uptake. Whether HA2 peptides interact with the endosome membrane after internalisation is key to its destabilisation and disruption, and a further cytosolic release of the particles. The quantity of HA2 peptides present within the nanoparticle monolayer can therefore have a direct influence on the efficiency of the endosomal membrane fusion. Increasing the number of HA2 function within each nanoparticle monolayer could increase the chances of endosomal disruption upon endosome acidification and of subsequent release of gold nanoparticles in the cell cytosol. To test this hypothesis, gold nanoparticles were prepared with higher proportions of HA2 peptides (50% and 100% mole/mole) within their self-assembled monolayer and compared with the behaviour observed previously for 10% HA2 (Fig. 2A-B). HeLa cells were incubated for 4h with nanoparticles (10nm diameter, 6nM) functionalised with either 50% CCALNN-dHA2 and 50% CALNN or 100% CCALNN-dHA2, fixed and imaged by TEM. The first important observation that can be drawn from the TEM images is that the presence of nanoparticle in the cell cytosol remains a rare event, regardless of the proportion (10%, 50% or 100%) of CCALNN-dHA2 peptides introduced within the nanoparticle monolayer. The TEM images suggest that more nanoparticles are present in endosomes in case of particles functionalisation with 50% HA2 (Fig. 3A, dataset available on figshare [70]) or 100% HA2 (Fig. 3B, dataset available on figshare [71]), as compared to 10% HA2 (Fig. 2A-B). However, it was not translated into a more efficient release of the particles from the endosomes. A quantification of the density of nanoparticles in endosomes was performed using the TEM images (Fig. 3C), as described earlier for Fig. 2E. A non-parametric statistical analysis of the three population variances (Kruskal-Wallis test) demonstrated that they are significantly different (p = 3.04 × 10^-3^). Further series of two-sample non-parametric tests (Mann-Whitney U) showed a significant difference of the density of nanoparticle in endosomes between the 10% HA2 and 50% HA2 samples (p = 0.043), between the 50% HA2 and 100% HA2 samples (p = 0.036) and between the 10% HA2 and 100% HA2 samples (p = 0.002). However, despite a higher number of CCALNN-dHA2 peptides available to disrupt the endosomes (*e*.*g*. for 100% CCALNN-dHA2), with more nanoparticles and more HA2 per nanoparticle in endosomes, the quantity of nanoparticles present in the cell cytosol did not increase accordingly and remained negligible. To explain the failure to release the nanoparticles from endosomes, various hypotheses can be formulated. The conformational change of HA2 necessary for membrane destabilisation could be hampered by HA2 molecular crowding at the nanoparticle surface, especially in the case of high percentage of HA2 coverage or of domain formation. Alternatively, the nanoparticles could also remain bound to the membrane through the HA2 fusion sequence following a partial or unsuccessful destabilisation that would in most case not be sufficient for a cytosolic release. The latter hypothesis is supported by a report by Lee *et al*. who observed endosome disruption by HA2 but no release of the HA2 analogue E5 fused to TAT-mCherry within the cytosol [49].

### Localisation of internalised HA2-capped nanoparticles: effect of the HA2 termini orientation

**Fig 4:**
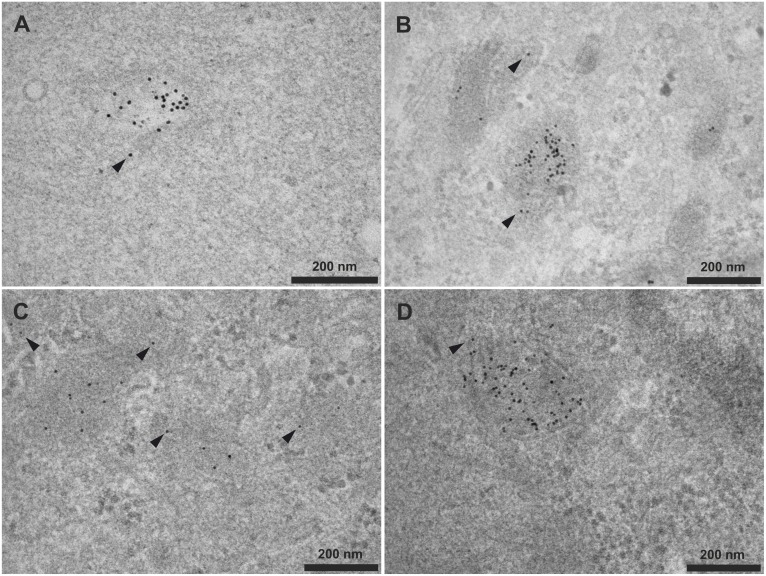
Attachment of HA2 to gold cores via the C-terminus versus N-terminus. HeLa cells were incubated for 4h with 10nm gold nanoparticles (6nM) capped with peptide monolayers of different compositions, fixed, and imaged by electron microscopy. Nanoparticle peptide monolayer compositions were: (A) 20% CCALNN-HA2 and 80% CALNN; (B) 20% HA2-NNLACC and 80% CALNN; (C) 50% CCALNN-HA2 and 50% CALNN; (D) 50% HA2-NNLACC and 50% CALNN. Arrowheads indicate particles around or outside endosomes. Additional images are available on figshare (10.6084/m9.figshare.874219, 10.6084/m9.figshare.874153, 10.6084/m9.figshare.874033, 10.6084/m9.figshare.873852).

The exact process by which HA2 destabilises membranes is not fully understood. This stems from a lack of precise structural data for the critical part of the HA2 sequence that undergoes conformational changes during the membrane fusion, due to failures at crystallising it (very high hydrophobicity at acidic pH) [41]. Han *et al*. identified the N–terminal domain of HA2 as critical for destabilisation of artificial model membranes. It was shown that all 20 residues of the fusion domain of HA2 were actively involved in the conformational changes necessary to insert deep into synthetic lipid bilayer membrane at acidic pH [42]. This process could be affected by the way the peptide is attached to the gold nanoparticle surface, e.g. attached to the gold core *via* either the N- or the C-terminal end. Two orientations of the HA2 peptide sequence (Table 1, sequences 2–3) were therefore compared to assess the influence of the direction of attachment to the gold core. These two sequences of HA2 peptide differ marginally from the sequence of HA2 peptide used previously (Table 1, sequence 1), but not enough to prevent the fusion process. Indeed, both sequences have been reported to show a consistent fusion activity (see Table A in S1 File for a comparison with other sequences from the literature) [38,39]. The HA2 fusion sequence was attached to the linker GGG by either its N-terminal end for the peptide designated as HA2-NNLACC, or via its C-terminal end for CCALNN-HA2 (Table 1, sequences 2,3). Nanoparticles were functionalised with a mixture of capping peptides CALNN and one of either version of the HA2 peptides. Two ratios (4:1 and 1:1) of the CALNN: CCALNN-HA2 or CALNN: HA2-NNLACC peptides were used to form the gold nanoparticles self-assembled monolayers. HeLa cells were then incubated separately with one of the resulting four types of nanoparticles (10nm diameter, 6nM) for 4h, fixed and imaged by TEM. The electron microscope images presented in Fig. 4A-D (datasets available on figshare [72–75]) do not show significantly different behaviours. Indeed, neither of the attachment orientations of the HA2 peptide showed a cytosolic release of the nanoparticles (compare Fig. 4A to Fig. 4B and Fig. 4C to Fig. 4D), with most of the particles inside endosomes or at their vicinity. For both orientations, varying the quantity of HA2 peptides present on the gold core did not show an improvement of the quantity of free cytosolic nanoparticles. Overall, attaching the HA2 peptides onto the gold nanoparticles surface by its N- or C-terminal end, with a larger (50%) or a smaller (20%) quantity of HA2 functions per nanoparticle, does not provoke any change in terms of cytosol localisation. The nanoparticles remain in intact endolysosomes or near ruptured ones.

## Conclusions

A quantitative analysis demonstrated that peptide-capped gold nanoparticles including either or both of TAT and HA2 peptides within their self-assembled monolayer have an increased cellular uptake. However, they remained inside endosomes or inserted into and attached to endolysosome membranes, and almost never escaped the vesicles. Addition of PEG in the monolayer to reduce non-specific interactions did not help separation from the membrane and did not change the vesicular localisation. Similarly, increasing the molar percentage of HA2 peptides within the monolayer resulted in an increased number of nanoparticles within endolysosomes, but did not improve the release to the cell cytosol. Varying the direction of attachment of HA2 peptides to the gold cores did not provide more favourable interactions of HA2 with the membrane following endocytosis, with nanoparticles remaining in both cases attached to the membrane.

## Supporting Information

S1 Fig Extinction spectra of peptide capped gold nanoparticles. 5 nm diameter gold nanoparticles were prepared and coated as indicated in the methods section. All coated particles were functionalised with a 4:1 ratio of CALNN: CCALNN-PEG (capping) peptides; 10%dHA2 had 10% of CCALNN-dHA2; 1% TAT had 1% of CALNN-TAT; 10%dHA2–1% TAT had 10% of CCALNN-dHA2 and 1% of CALNN-TAT; 10% TAT had 10% of CALNN-TAT. (A) UV-visible region. (B) Zoom from A on the plasmonic region to highlight the small shift of the plasmon band upon peptide-capping. The position of the heating laser used in our photothermal microscopy set up is indicated. A much larger shift indicative of aggregation is observed for particles with 10% TAT.
(TIF)

S2 Fig Photothermal microscope scheme. Photothermal, bright field and wide field fluorescence microscopy are combined on a single instrument. WL: white light, Ex.F: excitation filter (fluorescence), Em.F: emission filter (fluorescence), AOM: acousto-optic modulator, CM: cold mirror, PD: photodiode, RPF: redpass filter. Adapted from Cesbron (reference 76 available in S1 File).
(TIF)

S3 Fig Gold nanoparticles uptake decreases with increasing percentages of CCALNN-PEG in their monolayer. HeLa cells were incubated in suspension with 5nm diameter gold nanoparticles (final concentration 100nM) coated with a mix of CALNN and CCALNN-PEG peptides for 10 min in serum-free medium and a further 30 min in complete medium (10% FCS). Nanoparticles/medium were then discarded, cells transferred to a dish and left to attach for 4h in complete medium, fixed and later imaged by photothermal microscopy. (A-D) Photothermal microscopy images of the internalised nanoparticles. (A) 100% CALNN–0% CCALNN-PEG. (B) 90% CALNN–10% CCALNN-PEG. (C) 80% CALNN–20% CCALNN-PEG. (D) 70% CALNN–30% CCALNN-PEG. (E) Quantification of single cells mean photothermal intensities (∼50 cells per condition) for the four monolayer compositions shown in A-D. ∗ shows a statistical difference (one-way ANOVA test followed by a Holm-Bonferroni test) between pairs of conditions with non-matching bar colours (p < 0.01), with no significant difference between the pairs of the same colour. Error bars represent the SE. Scale bars represent 20μm. Adapted from Cesbron (reference 76 available in S1 File).
(TIF)

S4 Fig HA2-fused TAT peptides influence the intracellular delivery of FITC-TAT peptides. HeLa cells were incubated with the indicated peptides in medium for 5h. The cells were washed with warm 1 x PBS and fresh medium was added. The cells were then imaged by confocal laser scanning microscopy. (A–C) 10μM FITC-TAT peptides, (D–F) 10μM FITC-TAT and 2μM HA2-TAT peptides. Adapted from Shaheen (reference 77 available in S1 File).
(TIF)

S5 Fig Entry mechanism and localisation of gold nanoparticles capped with both CALNN-TAT and thiol-PEG. HeLa cells were incubated with 15nm gold nanoparticles (6nM) capped with 5% CALNN-TAT and 95% thiol-PEG (mole/mole) and washed thoroughly with 1x PBS before fixation and TEM imaging. (A-B) 10 min incubation time, (C-F) 2h incubation time, (G-H) 24h incubation time.
(TIF)

S1 File Supporting Information. Contains supporting tables Table A and Table B, supporting materials and methods, supporting discussions and supporting references.
(PDF)
