# Supplementary figures and images for: TAT and HA2 Facilitate Cellular Uptake of Gold Nanoparticles but Do Not Lead to Cytosolic Localisation
Source: PLoS One. 2015 Apr 2;10(4):e0121683. doi: 10.1371/journal.pone.0121683 (PMC4383524; doi:10.1371/journal.pone.0121683)

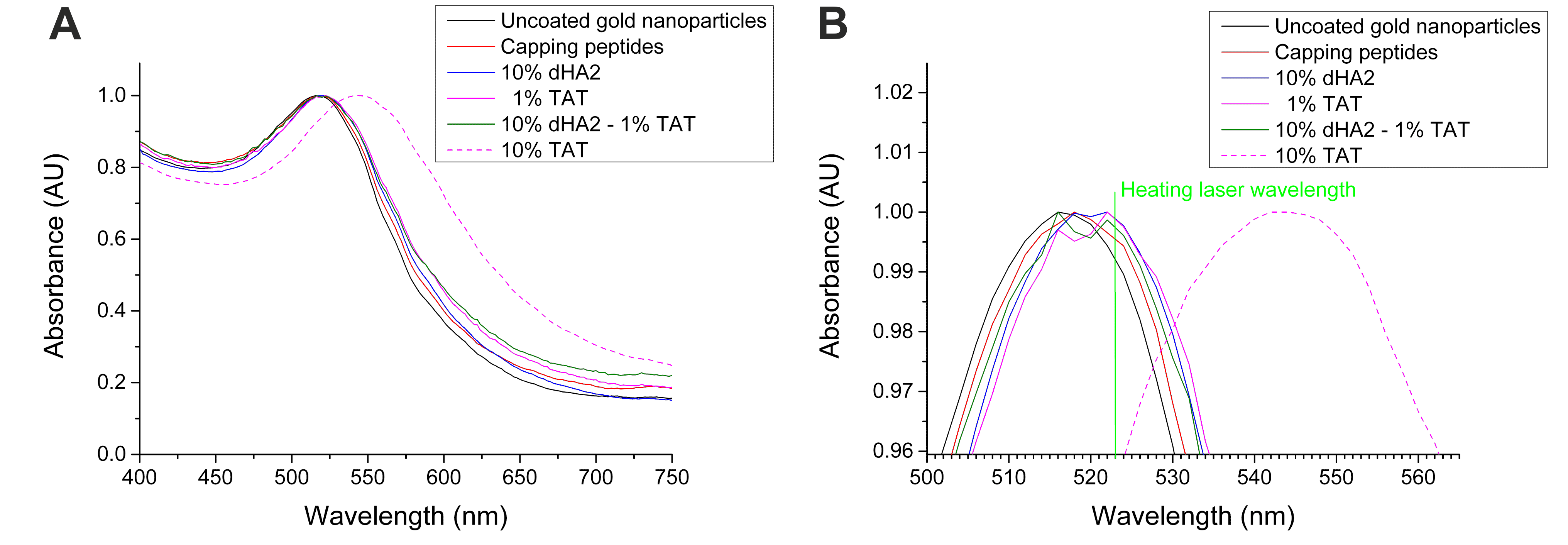

Supplement: S1 Fig — 5 nm diameter gold nanoparticles were prepared and coated as indicated in the methods section. All coated particles were functionalised with a 4:1 ratio of CALNN: CCALNN-PEG (capping) peptides; 10%dHA2 had 10% of CCALNN-dHA2; 1% TAT had 1% of CALNN-TAT; 10%dHA2–1% TAT had 10% of CCALNN-dHA2 and 1% of CALNN-TAT; 10% TAT had 10% of CALNN-TAT. (A) UV-visible region. (B) Zoom from A on the plasmonic region to highlight the small shift of the plasmon band upon peptide-capping. The position of the heating laser used in our photothermal microscopy set up is indicated. A much larger shift indicative of aggregation is observed for particles with 10% TAT. (TIF) [file pone.0121683.s001.tif]

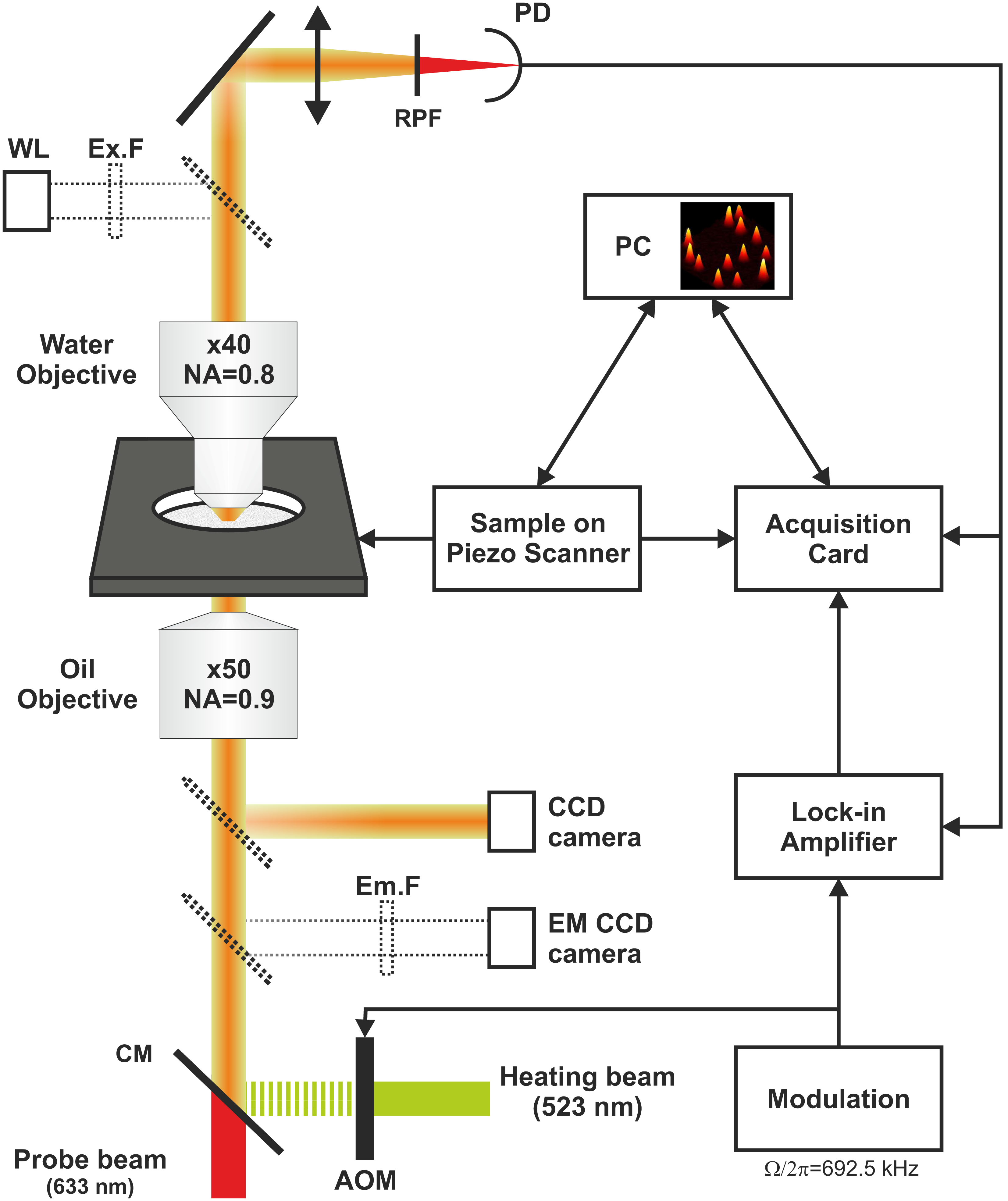

Supplement: S2 Fig — Photothermal, bright field and wide field fluorescence microscopy are combined on a single instrument. WL: white light, Ex.F: excitation filter (fluorescence), Em.F: emission filter (fluorescence), AOM: acousto-optic modulator, CM: cold mirror, PD: photodiode, RPF: redpass filter. Adapted from Cesbron (reference 76 available in S1 File). (TIF) [file pone.0121683.s002.tif]

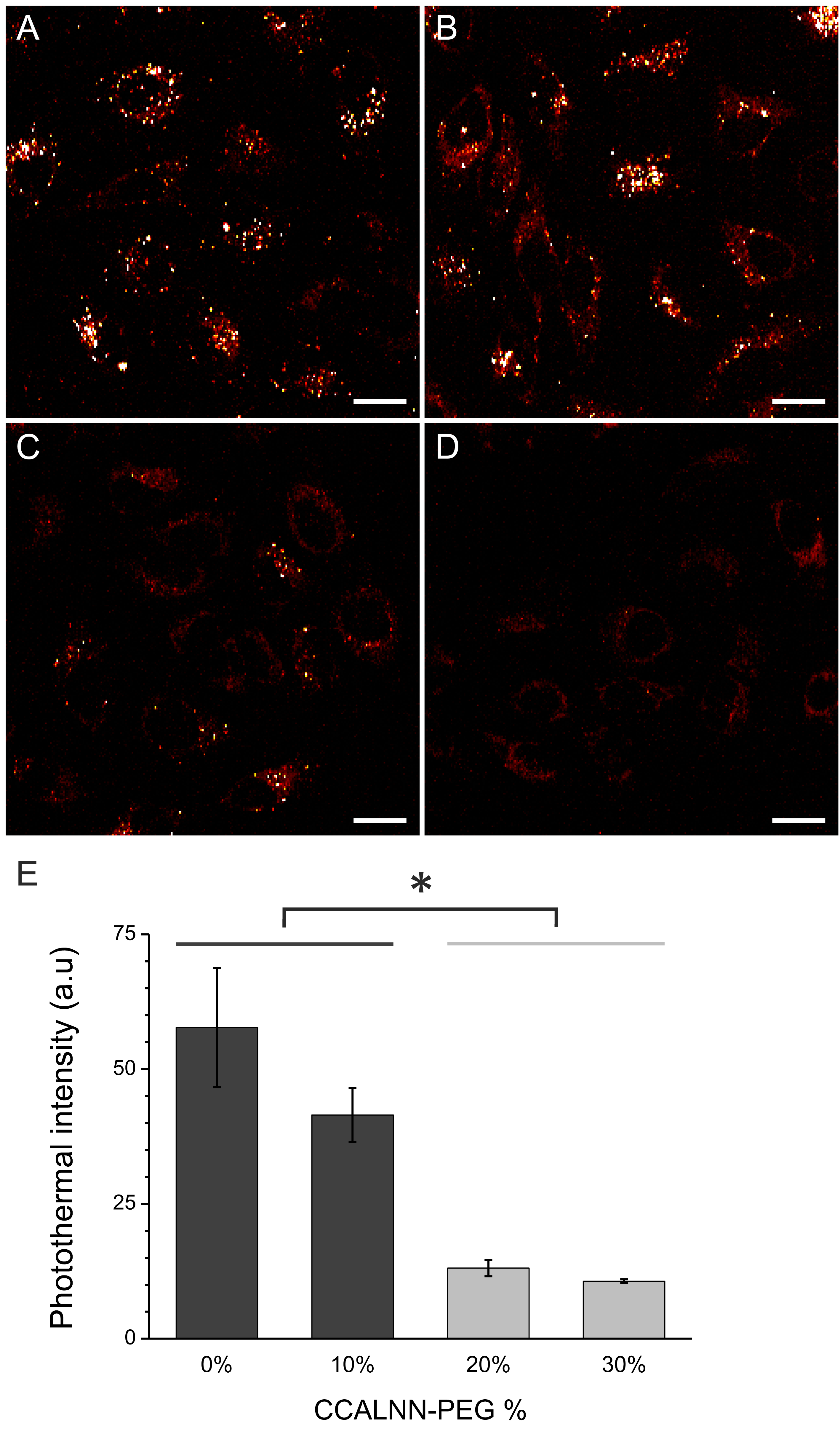

Supplement: S3 Fig — HeLa cells were incubated in suspension with 5nm diameter gold nanoparticles (final concentration 100nM) coated with a mix of CALNN and CCALNN-PEG peptides for 10 min in serum-free medium and a further 30 min in complete medium (10% FCS). Nanoparticles/medium were then discarded, cells transferred to a dish and left to attach for 4h in complete medium, fixed and later imaged by photothermal microscopy. (A-D) Photothermal microscopy images of the internalised nanoparticles. (A) 100% CALNN–0% CCALNN-PEG. (B) 90% CALNN–10% CCALNN-PEG. (C) 80% CALNN–20% CCALNN-PEG. (D) 70% CALNN–30% CCALNN-PEG. (E) Quantification of single cells mean photothermal intensities (∼50 cells per condition) for the four monolayer compositions shown in A-D. ∗ shows a statistical difference (one-way ANOVA test followed by a Holm-Bonferroni test) between pairs of conditions with non-matching bar colours (p < 0.01), with no significant difference between the pairs of the same colour. Error bars represent the SE. Scale bars represent 20μm. Adapted from Cesbron (reference 76 available in S1 File). (TIF) [file pone.0121683.s003.tif]

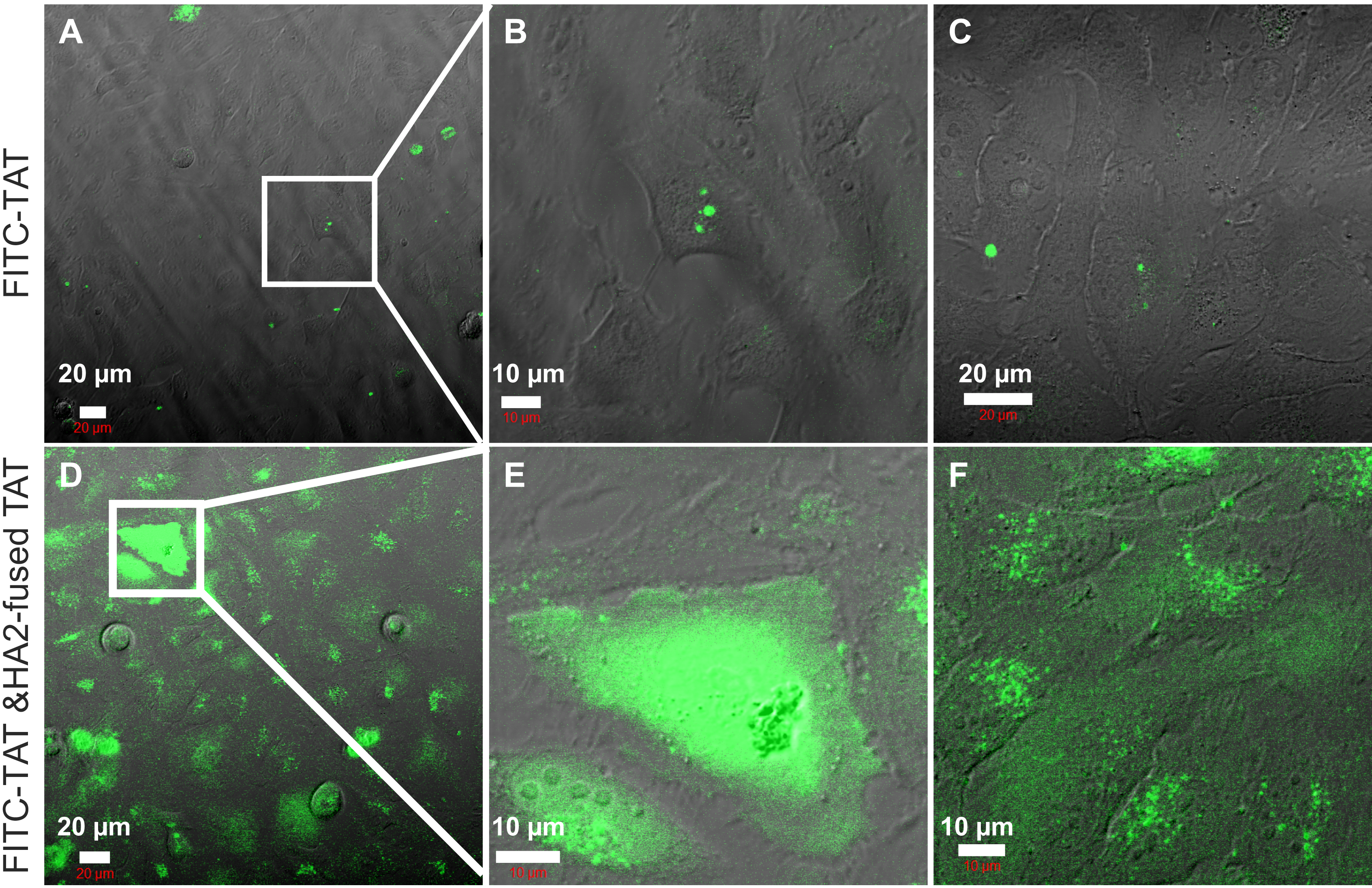

Supplement: S4 Fig — HeLa cells were incubated with the indicated peptides in medium for 5h. The cells were washed with warm 1 x PBS and fresh medium was added. The cells were then imaged by confocal laser scanning microscopy. (A–C) 10μM FITC-TAT peptides, (D–F) 10μM FITC-TAT and 2μM HA2-TAT peptides. Adapted from Shaheen (reference 77 available in S1 File). (TIF) [file pone.0121683.s004.tif]

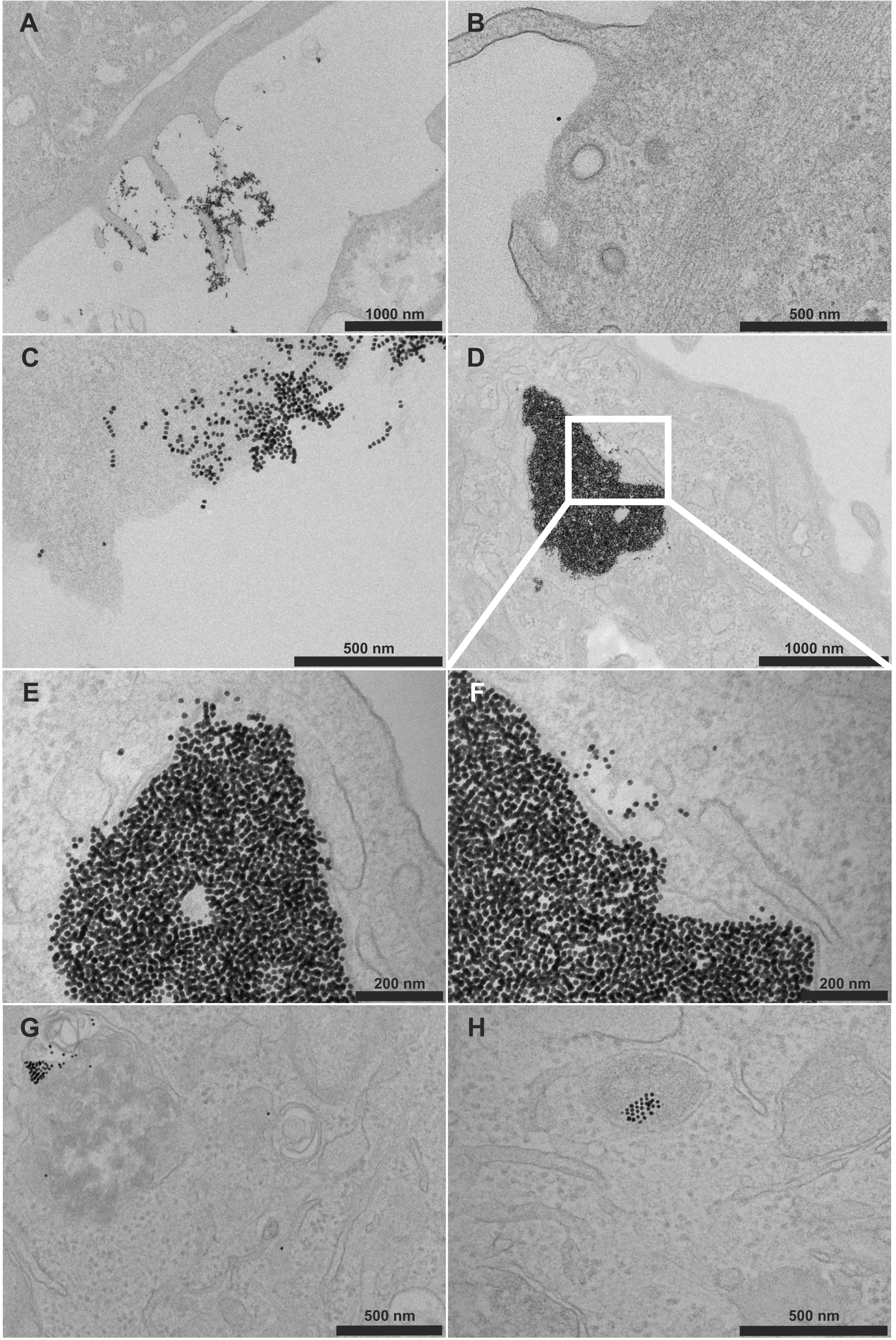

Supplement: S5 Fig — HeLa cells were incubated with 15nm gold nanoparticles (6nM) capped with 5% CALNN-TAT and 95% thiol-PEG (mole/mole) and washed thoroughly with 1x PBS before fixation and TEM imaging. (A-B) 10 min incubation time, (C-F) 2h incubation time, (G-H) 24h incubation time. (TIF) [file pone.0121683.s005.tif]
