# Supplementary material for: TAT and HA2 Facilitate Cellular Uptake of Gold Nanoparticles but Do Not Lead to Cytosolic Localisation
Source: PLoS One. 2015 Apr 2;10(4):e0121683. doi: 10.1371/journal.pone.0121683 (PMC4383524; doi:10.1371/journal.pone.0121683)
Supplement: S1 File — Contains supporting tables Table A and Table B, supporting materials and methods, supporting discussions and supporting references. (PDF) [file pone.0121683.s006.pdf]

Supporting Information S1 File

TAT and HA2 Facilitate Cellular Uptake of Gold Nanoparticles but do not Lead to Cytosolic Localisation

Yann Cesbron[1,2,3,4§], Umbreen Shaheen[2§], Paul Free[2,5], Raphaël Lévy [2*]

1 Department of Chemistry, University of Liverpool, Liverpool L69 7ZD, UK.

2 Institute of Integrative Biology, University of Liverpool, Liverpool L69 7ZB, UK.

3 CNRS, UMR 6290, Institute of Genetics and Development of Rennes, 35043 Rennes, France.

4 Université de Rennes 1, Université Européenne de Bretagne, Structure fédérative de recherche Biosit, Faculté de Médecine, 35043 Rennes, France.

5 Institute of Materials Research and Engineering, A*STAR, 3 Research Link, Singapore 117602, Singapore.

§These authors contributed equally to this work.

* rapha@liverpool.ac.uk

Table of Contents

Table A	Nomenclature and sequences of the ligands mentioned in the literature	2
	**Materials and methods for supplementary figures**	2
Table B	Nomenclature of the ligands utilised	2
	**Increasing PEG in gold nanoparticles SAMs decreases cellular uptake of gold**	3
	**FITC-TAT cellular uptake in presence of HA2-fused TAT**	3
	**Macropinocytotic entry of TAT and PEG functionalised gold nanoparticles**	3
	**References**	4

Supporting Information S1 File

Table A. Nomenclature and sequences of the ligands mentioned in the literature. The sequences are taken as described in the articles cited in the references, whenever available. * indicates a sequence accessed on the author website (details in reference), and † a sequence inferred from other sequences in the paper.

Construct name	Construct sequence	Reference
CALNN–TAT	CALNNAGRKKRRQRRR	This ms
CALNN-TAT	QPPRRRQRRKKRGNNLAC	Todorova *et al.* [78]
TAT–Cre *	GRKKRRQRRRGHMASMTGGQQMGRDPNS–Cre	Wadia *et al.* [79,80]
dTAT	RRRQRRKKRG	Wadia *et al.* [79]
Biotin–TAT †	Biotin-CKYGRRRQRRKKRG-OH	Kumar *et al.* [81]
FITC-TAT	YGRKKRRQRRRK-FITC	Sugita *et al.* [82], this ms (SI)
TAT	GRKKRRQRRR	Lee *et al.* [83]
Unmodified HA2	GLFGAIAGFIENGWEGMIDGWYG	Cross *et al.* [84]
CCALNN–HA2	CCALNNGGGGLFEAIEGFIENGWEGMIDGWYG	This ms
HA2	GLFEAIEGFIENGWEGMIDGWYG	Liou *et al.* [85]
CCALNN–dHA2	CCALNNGdimGewGneifGaiaGflG-NH_2	This ms
dHA2	GDIMGEWGNEIFGAIAGFLG	Wadia *et al.* [79]
dTAT–HA2	RRRQRRKKRGGDIMGEWGNEIFGAIAGFLG	Wadia *et al.* [79]
Biotin–TAT–HA2–OH	Biotin-CKYGRRRQRRKKRGGDIMGEWGNEIFGAIAGFLG-OH	Kumar *et al.* [81]
pR9–HA2–mCherry	T7-6His-RRRRRRRRR-GLFEAIEGFIENGWEGMIDGWYG-mCherry	Liou *et al.* [85]
HA2–TAT	GLFEAIEGFIENGWEGMIDGWYGYGRKKRRQRRR	Sugita *et al.* [82], this ms (SI)
HA2–p53–9R	GLFEAIEGFIENGWEGMIDGWYG-p53-RRRRRRRRR	Michiue *et al.* [86]
E5–TAT–mCherry	GLFEAIAEFIENGWEGLIEGWYGGRKKRRQRRR-mCherry	Lee *et al.* [83]

Materials and methods for supplementary figures

HA2-TAT and FITC-TAT peptides (Table B, sequences 1 & 3) were purchased from Peptides Proteins Research Ltd (UK), Thiol-PEG (Table B, sequence 4) was from ProChimia (Poland). 1 % (w/v) poly-L-lysine solution was purchased from Sigma-Aldrich. The remaining peptides and reagents are as described in the main manuscript. A 100 mM solution of 11-mercaptoundecyl-tetra ethylene glycol (HS-$(CH_2)_{11}$-$(EG)_4$-ol) was prepared in methanol. The HA2-TAT and FITC-TAT peptides were dissolved in Milli-Q water at stock concentrations of 1 mg/mL. The resulting solutions were aliquoted and stored at –80 °C.

Table B. Nomenclature of the ligands utilised.

Sequence No.	Ligand name	Ligand sequence
1	HA2–TAT	GLFEAIEGFIENGWEGMIDGWYGYGRKKRRQRRR
2	CALNN–TAT	CALNNAGRKKRRQRRR
3	FITC–TAT	YGRKKRRQRRRK-fluorescein isothiocyanate
4	Thiol–PEG	HS-$(CH_2)_{11}$-(ethylene glycol)$_4$-glycinol

Supporting Information S1 File

Increasing PEG in gold nanoparticles SAMs decreases cellular uptake of gold

In order to gain insights on the effect of poly-(ethylene-glycol) on cellular uptake of gold nanoparticles, the following four peptide monolayers were designed with increasing percentages of PEG to coat the gold nanoparticles (5 nm diameter):

- 100% CALNN, 0% CCALNN-PEG (0%)
- 90% CALNN, 10% CCALNN-PEG (10%)
- 80% CALNN, 20% CCALNN-PEG (20%)
- 70% CALNN, 30% CCALNN-PEG (30%)

HeLa cells were incubated in suspension with gold nanoparticles (100 nM) for 40 minutes (10 min in serum-free medium and a further 30 min in complete medium (10 % FCS)), before the gold nanoparticles were removed by centrifugation. The cells were then seeded on Iwaki glass coverslip bottom dishes and left for 4 h to attach to the dish before fixation. The fixed cells were kept in 1x PBS at 4 °C with 0.05 % (w/v) sodium azide until photothermal imaging (S3 Fig, dataset available on figshare [87]).

FITC-TAT cellular uptake in presence of HA2-fused TAT

HA2-TAT and FITC-TAT peptides were used to assess the effect of HA2-fused TAT in disrupting endosomes and the subsequent release of the FITC-TAT into the cytosol as reported by Sugita *et al.* [82].

HeLa cells (4 x 10^5 cells/dish) were grown overnight in glass coverslip bottom dishes. The coverslip surface was coated with the positively charged polymer poly–L–lysine (1 %), in order to later minimise the adherence of positively charged TAT or HA2-fused TAT peptides and consequently maximise their availability to cellular uptake. The peptides were then incubated with cells for 5 h in medium (DMEM with 10% FBS and 1% NEAA). The cells were washed thoroughly three times with warm 1x PBS to remove the peptide-containing medium, before addition of fresh medium (37 °C). The cells containing dishes were then incubated on the microscope stage at 37 °C and 5 % CO_2, and observed by laser scanning confocal microscopy using a Zeiss LSM510 with a Plan-apochromat 40× or 63× oil immersion Zeiss objective. Excitation of fluorescein was performed using an argon ion laser at 488 nm. The emitted light was detected through a 500–550 nm band pass filter after a 490 nm dichroic mirror. Data capture was carried out with LSM510 version 3 software (Zeiss, Germany).

An increased cellular uptake of FITC-TAT was observed in the presence of HA2-fused TAT peptides suggesting that HA2-fused TAT cause an increased cellular uptake of FITC-TAT (S4 Fig). This contrasts with the report from Sugita *et al.* [82], but is in agreement with Liou *et al.* [85]. While uptake increased, the intracellular localisation did not suggest a cytosolic delivery of FITC-TAT. Indeed, only a small number of cells showed a diffuse FITC-TAT cytosolic signal (S4 Fig D&E).

Macropinocytotic entry of TAT and PEG functionalised gold nanoparticles

15 nm gold nanoparticles were prepared from a 5 % CALNN-TAT and 95 % thiol-PEG (mole/mole) combination of ligands (Table B, sequences 2 and 4) [88]. No nanoparticles were found inside the HeLa cells. It was hypothesized that the formed monolayer may not have reflected the mixture of ligands with little or no incorporation of the peptide ligands.

In order to increase the chance of an even distribution of TAT and PEG on the gold core surface a second method, adapted from Duchesne *et al.* [89], was used to functionalise the gold nanoparticles with the same CALNN-TAT and thiol-PEG ligands. Both stock solutions of CALNN-TAT (0.7 mM in DMSO) and thiol-PEG (100 mM in methanol) were diluted by factor of 6 in ethanol, before mixing them together, in order to increase the solubility of the mixture. The final percentage of ligands per gold nanoparticle was kept identical, *i.e.* 5 % CALNN-TAT peptides and 95 % thiol–PEG (mole/mole). The diluted and mixed CALNN-TAT peptide and thiol-PEG solution was poured into the colloidal gold nanoparticles solution, which was already under a vigorous stir (magnetic stirring plate). Tween-20 was added to a final concentration of 0.05 % (v/v) afterward and the resulting solution was stirred overnight at room temperature. The 100 % PEG-capped gold nanoparticles were prepared the same way. The purification/washing procedure for 15 nm gold nanoparticle was the same as described for 10 nm gold nanoparticles (experimental section of the main manuscript) with a centrifugation time reduced to 15 min. The gold nanoparticles functionalised with 5 % CALNN-TAT and 95 % thiol-PEG (mole/mole) were incubated with HeLa cells in medium for 10 min, 2 h, 24 h or 48 h.

TEM examination showed that gold nanoparticles were taken up mainly by macropinocytosis (S5 Fig, datasets available on figshare [90-95]). Cell membrane extensions were observed with large numbers of associated gold nanoparticles (S5 Fig A-F). Macropinocytosis could explain why a large uptake of gold nanoparticles functionalised with thiol-PEG occurred.

Supporting Information S1 File

References

76. Cesbron Y. Intracellular delivery and fate of peptide-capped gold nanoparticles. PhD Thesis, University of Liverpool. 2011.

77. Shaheen U. Delivery, interaction and fate of peptide-capped gold nanoparticles in mammalian cells. PhD Thesis, University of Liverpool. 2012.

78. Todorova N, Chiappini C, Mager M, Simona B, Patel II, Stevens MM, et al. Surface Presentation of Functional Peptides in Solution Determines Cell Internalization Efficiency of TAT Conjugated Nanoparticles. Nano Letters. 2014; 14: 5229-5237.

79. Wadia JS, Stan RV, Dowdy SF. Transducible TAT-HA fusogenic peptide enhances escape of TAT-fusion proteins after lipid raft macropinocytosis. Nature Medicine. 2004; 10: 310-315.

80. Wadia JS, Dowdy SF (13 Nov 2004) pET 28.2 TAT-Cre vector sequence. Available: http://cmm.ucsd.edu/Lab_Pages/dowdy/Vectors/vector_seq/28.2%20TAT-Cre.pdf. Accessed 24 February 2015.

81. Kumar S, Harrison N, Richards-Kortum R, Sokolov K. Plasmonic nanosensors for imaging intracellular biomarkers in live cells. Nano Letters. 2007; 7: 1338-1343.

82. Sugita T, Yoshikawa T, Mukai Y, Yamanada N, Imai S, Nagano K, et al. Improved cytosolic translocation and tumor-killing activity of Tat-shepherdin conjugates mediated by co-treatment with Tat-fused endosome-disruptive HA2 peptide. Biochemical and biophysical research communications. 2007; 363: 1027-1032.

83. Lee Y-J, Johnson G, Peltier GC, Pellois J-P. A HA2-Fusion tag limits the endosomal release of its protein cargo despite causing endosomal lysis. Biochimica Et Biophysica Acta-General Subjects. 2011; 1810: 752-758.

84. Cross KJ, Langley WA, Russell RJ, Skehel JJ, Steinhauer DA. Composition and Functions of the Influenza Fusion Peptide. Protein and Peptide Letters. 2009; 16: 766-778.

85. Liou J-S, Liu BR, Martin AL, Huang Y-W, Chiang H-J, Lee H-J. Protein transduction in human cells is enhanced by cell-penetrating peptides fused with an endosomolytic HA2 sequence. Peptides. 2012; 37: 273-284.

86. Michiue H, Tomizawa K, Wei F-Y, Matsushita M, Lu Y-F, Ichikawa T, et al. The NH2 Terminus of Influenza Virus Hemagglutinin-2 Subunit Peptides Enhances the Antitumor Potency of Polyarginine-mediated p53 Protein Transduction. Journal of Biological Chemistry. 2005; 280: 8285-8289.

87. Cesbron Y, Shaheen U, Levy R. CCALNN-PEG monolayer proportion influence on gold nanoparticles uptake (Figure S3 dataset); 2014. Database: figshare [Internet]. http://dx.doi.org/10.6084/m9.figshare.1088399. Accessed 24 February 2015.

88. Krpetic Z, Saleemi S, Prior IA, See V, Qureshi R, Brust M. Negotiation of intracellular membrane barriers by TAT-modified gold nanoparticles. ACS Nano. 2011; 5: 5195-5201.

89. Duchesne L, Gentili D, Comes-Franchini M, Fernig DG. Robust ligand shells for biological applications of gold nanoparticles. Langmuir. 2008; 24: 13572-13580.

90. Shaheen U, Levy R. 10 min incubation with 5% CALNNTAT and 95% PEG capped gold nanoparticles; 2014. Database: figshare [Internet]. http://dx.doi.org/10.6084/m9.figshare.1246458. Accessed 19 November 2014.

91. Shaheen U, Levy R. 2 h incubation of HeLa cells with 5% CALNNTAT and 95% PEG capped gold nanoparticles; 2014. Database: figshare [Internet]. http://dx.doi.org/10.6084/m9.figshare.1246609. Accessed 19 November 2014.

92. Shaheen U, Levy R. 2 h incubation of HeLa cell with 100% PEG capped gold nanoparticles; 2014. Database: figshare [Internet]. http://dx.doi.org/10.6084/m9.figshare.1246622. Accessed 19 November 2014.

93. Shaheen U, Levy R. 24 h incubation of HeLa cells with 5% CALNNTAT and 95% PEG capped (mole/mole) capped gold nanoparticles; 2014. Database: figshare [Internet]. http://dx.doi.org/10.6084/m9.figshare.1246660. Accessed 19 November 2014.

94. Shaheen U, Levy R. 48 h incubation of HeLa cells with 5% CALNNTAT and 95% PEG (mole/mole) capped gold nanoparticles; 2014. Database: figshare [Internet]. http://dx.doi.org/10.6084/m9.figshare.1246696. Accessed 19 November 2014.

95. Shaheen U, Levy R. 48 h incubation of HeLa cells in medium only (a control); 2014. Database: figshare [Internet]. http://dx.doi.org/10.6084/m9.figshare.1246707. Accessed 19 November 2014.
